# Advances in *Campylobacter*: Molecular Epidemiology, Virulence Factors, Immune Responses and Drug Resistance

**DOI:** 10.3390/microorganisms12010028

**Published:** 2023-12-22

**Authors:** Andreas E. Zautner

**Affiliations:** 1 Institute of Medical Microbiology and Hospital Hygiene, Medical Faculty, Otto-von-Guericke University Magdeburg, 39120 Magdeburg, Germany; azautne@gwdg.de; 2 Health Campus Immunology, Infectiology and Inflammation (GCI), Medical Faculty, Otto-von-Guericke University Magdeburg, 39104 Magdeburg, Germany

*Campylobacter* infections, caused by *Campylobacter jejuni* and *Campylobacter coli*, are a major global concern, particularly as they are the leading cause of bacterial enteritis. However, there are also other *Campylobacter*-related organisms, such as *Arcobacter* species or facultative anaerobic *Campylobacter* species, that have not been extensively studied for their clinical relevance, virulence, pathogenesis, and antimicrobial resistance. In addition to causing acute infections, *Campylobacter* infections can lead to post-infectious sequelae, such as Guillain–Barré syndrome (GBS), which are linked to the complexity of the initial immune response against the bacteria [1].

To address these knowledge gaps, a Special Issue was initiated which focuses on the epidemiology, antibiotic susceptibility, proteomics, genomics, and virulence of *Campylobacter* and closely related microbial species. The scope of this Special Issue also includes the immunopathogenesis of post-infectious sequelae. Original research articles, review articles, and case reports, especially those involving rare *Campylobacter* species and genome data, are within this scope.

One of the challenges is the overdiagnosis and over-detection of *Campylobacter* species, particularly in food samples and in the monitoring of poultry farms. In addition to viable *Campylobacter* isolates, *Campylobacter* also exists in a state known as Viable but Non-Culturable (VBNC) [2]. As the name implies, VBNC forms are considered to be non-virulent, but with the general trend of replacing culture-based CFU determination with molecular biology techniques, there is a risk of over-detection and, consequently, the unnecessary application of decontamination measures, especially in poultry populations.

Another little-addressed diagnostic gap concerns the prevalence and virulence of the closest relatives of *Campylobacter*, the various *Arcobacter* species [3]. These are not routinely tested in most laboratories and are not included in most commercial multiplex assays. As a result, their contribution to gastroenteritis cases is currently completely underdiagnosed.

A further area in *Campylobacter* research that has been relatively unexplored so far is the subclinical infection of livestock such as chickens, turkeys, and swine by *Campylobacter* species, which are widely considered part of the normal flora of these animals but which can lead to reduced vitality and delayed weight gain in these livestock due to the disruption of the intestinal epithelial barrier [4,5,6]. This results in significant economic losses in food production.

And lastly, for a large portion of the proteins encoded in the *Campylobacter* genome, the function and, thus, the significance for pathogenesis and host colonization or adaptation to various habitat conditions remain unclear. Therefore, there continues to be a need for comparative and functional genomic and quantitative proteomic analyses [7].

In conclusion, this Special Issue on *Campylobacter* research provides valuable insights into the epidemiology, virulence factors, immune responses, and drug resistance of *Campylobacter* and related microbial species. The articles included in this Special Issue shed light on important aspects of *Campylobacter* infections and contribute to our understanding of these pathogens and their impact on human and animal health.
